# Supplementary figures and images for: SARS-CoV-2 evolution balances conflicting roles of N protein phosphorylation
Source: PLoS Pathog. 2024 Nov 21;20(11):e1012741. doi: 10.1371/journal.ppat.1012741 (PMC11620656; doi:10.1371/journal.ppat.1012741)

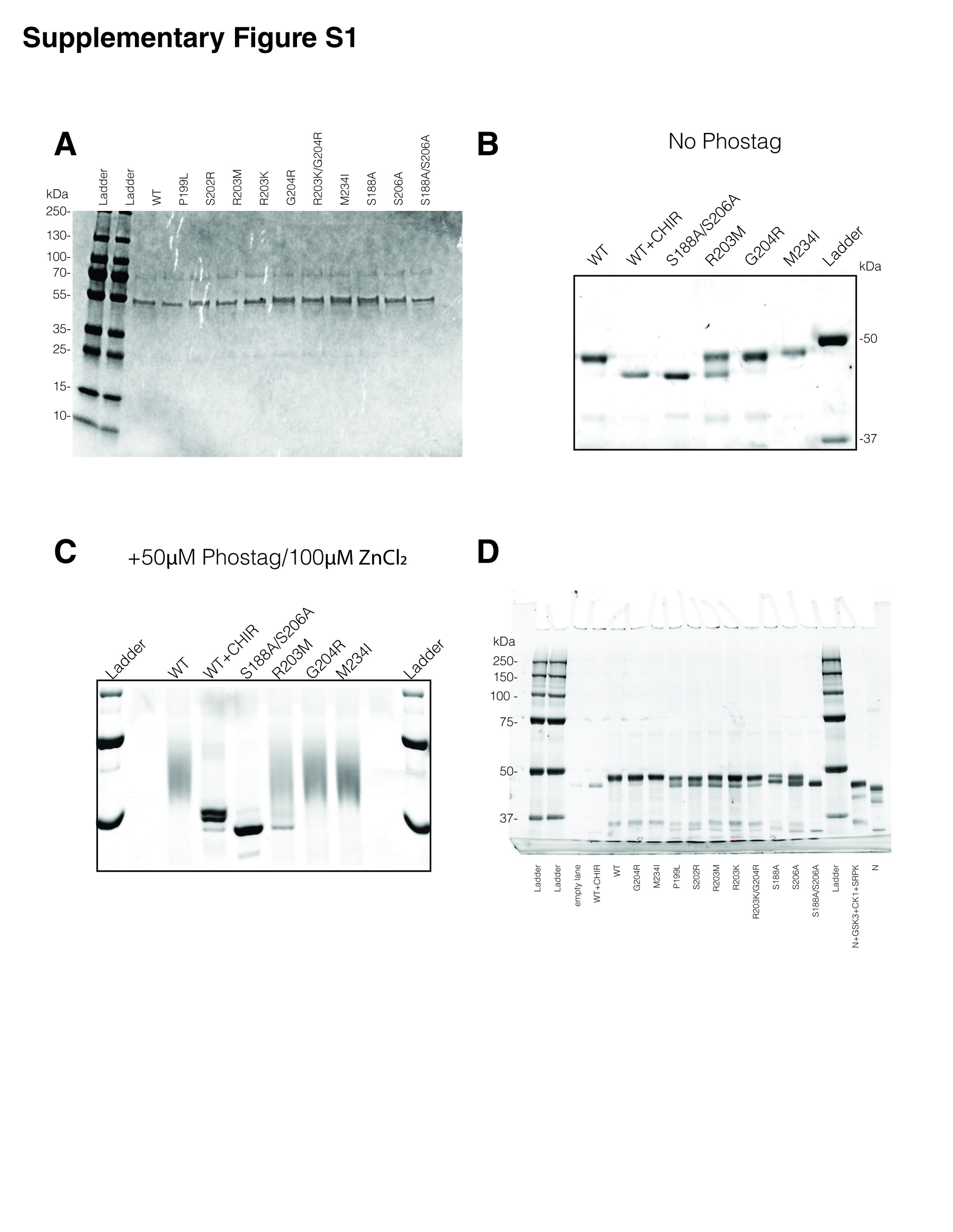

Supplement: S1 Fig — A) Coomassie blue stained gel image for the autoradiography image shown in Fig 1C. B) N protein variants affinity-purified from transfected 293T lysates visualized by Krypton staining on a 7.5% SDS-PAGE gel. CHIR indicates lysate purified from cells treated with 2.5 μM CHIR98014, a GSK-3 inhibitor. C) Same N protein variants visualized by Krypton staining on a PhosTag phosphate affinity gel. Note: molecular weight for ladder bands is not labeled since migration in PhosTag gels does not correspond directly with molecular weight. D) Full gel image for the gel used in Fig 1E showing several naturally occurring and engineered N protein variants isolated from 293T lysates. The last two lanes are N proteins isolated from E. coli with and without in vitro phosphorylation using GSK-3, CK1 and SRPK. (TIF) [file ppat.1012741.s001.tif]

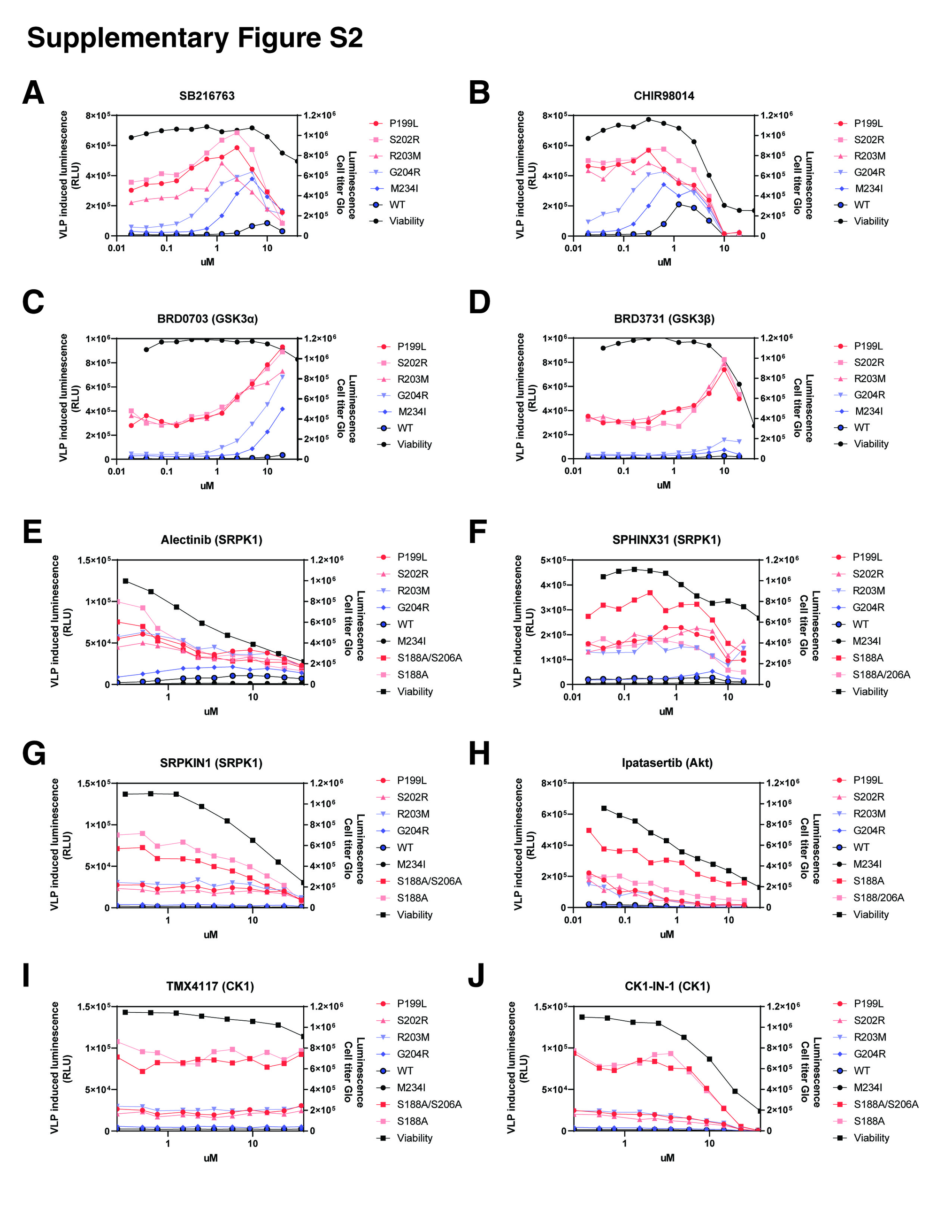

Supplement: S2 Fig — A-D) Left axis shows luminescence from cells treated with VLPs generated with N mutants: P199L, S202R, R203M, G204R, M234I and WT. Producer cells were treated with indicated concentrations (20 nM to 20 μM) of GSK-3 inhibitors: SB216763, CHIR98014, BRD0703, BRD3731. Right axis and black lines shows viability as measured by CellTiter-Glo luminescence from cells treated with the same concentrations of corresponding drugs but not with VLPs. E-H) Left axis shows normalized luminescence from cells treated with VLPs generated with N mutants: S188A, S188A/S206A, P199L, S202R, R203M, G204R, M234I and WT. Producer cells were treated with indicated concentrations (20 nM to 20 μM) of inhibitors targeting multiple kinases: Alectinib (SRPK1), SPHINX31 (SRPK1), SRPKIN1 (SRPK1), Ipatesertib (AKT), TMX4117 (CK1), CK1-in-1 (CK1). Right axis and black lines shows viability as measured by CellTiter-Glo luminescence from cells treated with the same concentrations of corresponding drugs but not with VLPs. No replicates were used for the data in these panels. (TIF) [file ppat.1012741.s002.tif]

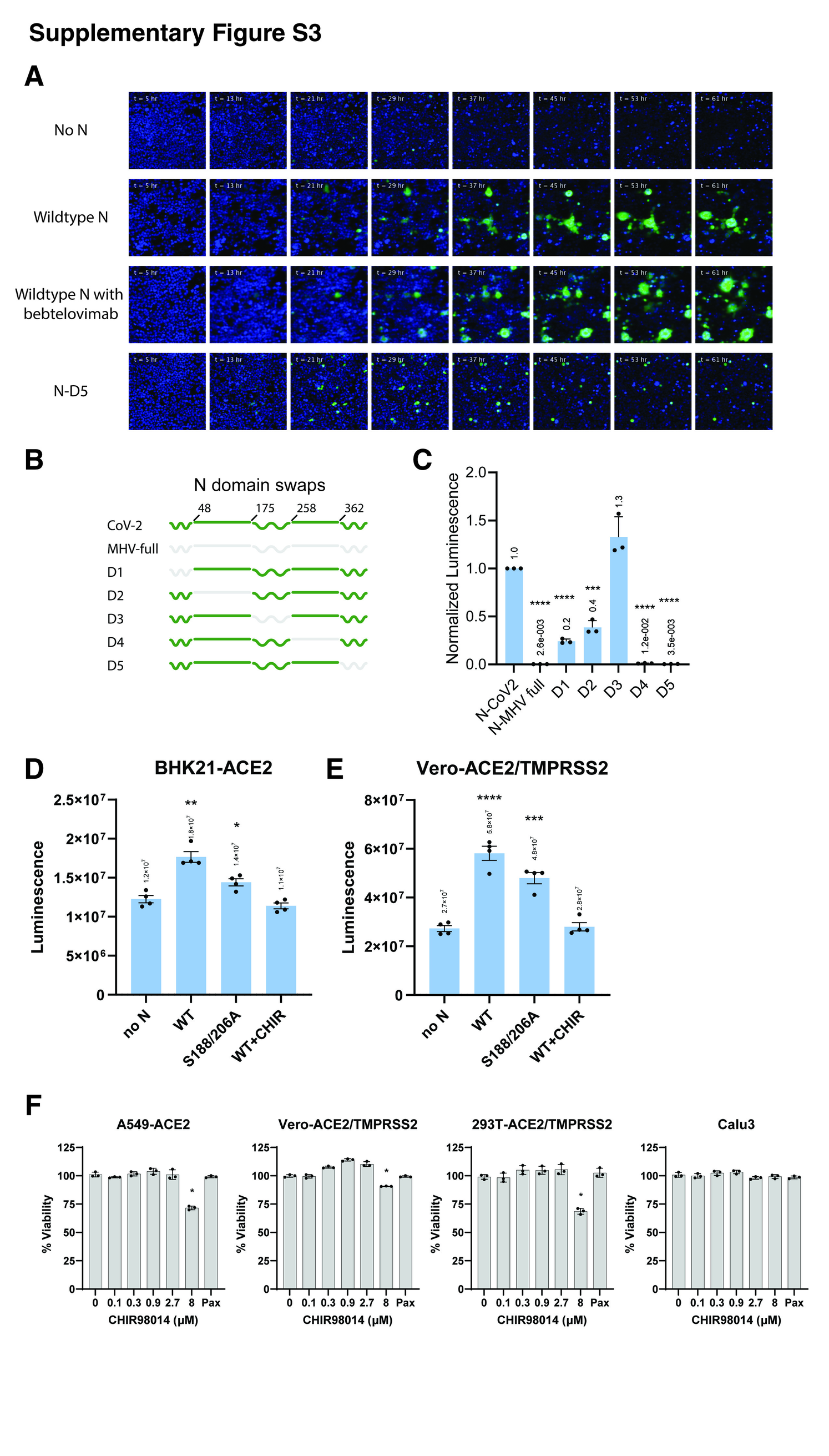

Supplement: S3 Fig — A) Live microscopy snapshots with nuclei shown in blue (Hoechst 34580) and GFP in green (eGFP, produced from infection) from SCIP infected cells (293T-ACE2/TMPRSS2). Cells were transfected with indicated N protein variant. Large green spots show infection of a cluster neighboring cells resulting from cell to cell spread. B) Schematic of domain swap variants of N proteins from SARS-CoV-2 and MHV-A59. Numbers indicate positions on SARS-CoV-2. C) Luminescence signal from VLP assay using domain swap N proteins. N-D5 has negligible signal indicating that it is packaging defective. D, E) Luminescence signal from SCIP infected BHK21-ACE2 (D) and VeroE6-ACE2/TMPRSS2 (E) that were transfected with N proteins with indicated mutations or treated with CHIR98014 at 3 μM. VeroE6-ACE2/TMPRSS2 were also treated with 2 μM of the P-glycoprotein efflux inhibitor CP-100356 due to their high endogenous efflux activity. The data are presented as mean +/- SD of one independent experiment conducted in three (C) and four (D and E) replicates. *, p<0.05; **, p<0.01; ***, p<0.001; ****, p<0.0001 by Student’s T-test. F) Cytotoxicity data of CHIR98014 in indicated cells corresponding to treatments in Fig 3E–3H. Data is presented as mean +/- SD of three independent replicates conducted in one technical replicate. *, p<0.05 by Student’s T-test compared to no treatment control. (TIF) [file ppat.1012741.s003.tif]

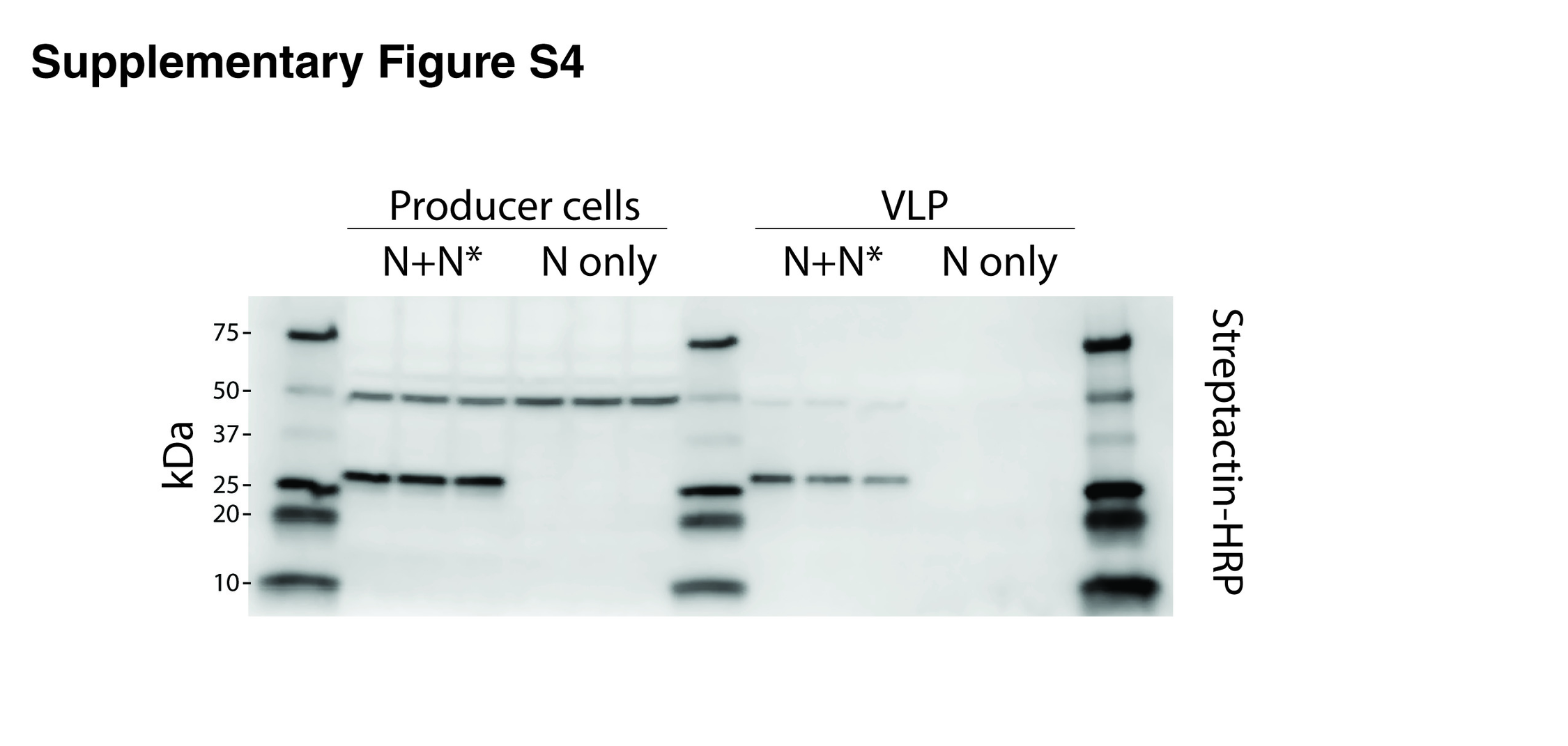

Supplement: S4 Fig — N* and N plasmids both contain C-terminal 2xStrepTag2 and are imaged using StreptactinXT-HRP. VLPs purified by ultracentrifugation. The blot is representative of one independent experiment conducted in triplicate. Quantification data are in Fig 5D. (TIF) [file ppat.1012741.s004.tif]
