# Supplementary material for: SARS-CoV-2 evolution balances conflicting roles of N protein phosphorylation
Source: PLoS Pathog. 2024 Nov 21;20(11):e1012741. doi: 10.1371/journal.ppat.1012741 (PMC11620656; doi:10.1371/journal.ppat.1012741)
Supplement: Attachment — Submitted filename: N-phosphorylation response to reviewers.docx [file ppat.1012741.s005.docx]

**Point-by-point response to reviewers’ comments**

**Part I - Summary**
Reviewer #1: Syed et al study the role of the N protein in the enhanced fitness of SARS-CoV-2 variants. They used single-cycle and virus-like particle assays to show that N phosphorylation in the central region of the protein has opposing effects on viral assembly and genome replication. The N protein from ancestral SARS-CoV-2 is highly phosphorylated, associated to high levels of genome replication but lower particle assembly compared to recent lineages. The authors also report on a new open reading frame, encoding a truncated N protein called N*, that occurs in the B.1.1 lineage and appears to support high levels of assembly and replication. N* improves viral fitness independently of phosphorylation. The results help explain the enhanced fitness of variants of concern.

We thank the reviewer for their supportive comments regarding the enhanced fitness of SARS-CoV-2 variants of concern that can now be linked to N protein phosphorylation.

Reviewer #2: This manuscript by Syed and colleagues describes the conflicting role of SARS-CoV-2 N protein phosphorylation in viral particle assembly and genome replication. It is an interesting study that provides insight into the pathogenesis of SARS-CoV-2.

We thank the reviewer for pointing out that our study defines a new role of N protein phosphorylation in the pathogenesis of SARS-CoV-2.

Reviewer #3: In this manuscript, Doudna and colleagues describe the impact of SARS-CoV-2 nucleocapsid (N) protein phosphorylation and mutation of the SR motif within the linker region found in variants on viral assembly and replication. The authors first assessed recombinant protein phosphorylation using 32P--ATP labeling and SDS-PAGE and show a decrease in phosphorylation for N proteins containing previously described mutations P199L, S202R, R203M, and R203K. They next test the impact of these mutations on viral assembly using a VLP luciferase assay and treatment with different GSK-3 or kinase inhibitors. The data show that reduced phosphorylation results in increased assembly and inhibition of GSK activity recovered VLP assembly. To test the impact of phosphorylation on replication, the authors developed a single cycle infection assay for SARS-CoV-2 and generated N SCIPs and found that increased N phosphorylation results in increased infection and growth rates that they relate to viral fitness. In addition, the authors evaluate the role of a low abundant, truncated N* protein that is found in the B.1.1. lineage and find that expression of N* appears to enhance viral assembly of VLPs and improve viral fitness. Overall, the studies described here appear to extend findings on the effect of N protein phosphorylation on SARS-CoV-2 replication and assembly. The coauthors previously analyzed the P199L, S202R, R203M, and R203K mutations present in variants of concern in a VLP assay and demonstrated an increase in VLP assembly (PMID: 34735219). However, two other studies also characterized the impact of enhanced phosphorylation of N protein with the evaluation of the S202R and R203K/G204R mutants (PMID: 36572560 and 35728038). They show that R203K/G204R increases phosphorylation of N and a corresponding increase in viral replication. While the authors here acknowledge that their findings are different, they do not provide additional rigorous experimental evidence to further bolster their model for N phosphorylation in replication. GSK-3 inhibition has also been shown in prior studies to impact N phosphorylation and the addition of other GSK-3 inhibitors in this study is complicated by effects of cytotoxicity. The one potentially interesting description here is the evaluation of a role for N* in assembly; however, studies here are limited by a lack of in-depth assessment of N*, including expression levels that would be relevant in infection and N* on the multiple roles N has in SARS-CoV-2 infection.

The reviewer points out two critical issues with our study relating to the discrepancy with other studies regarding S202R and R203K/G204R mutations as well as lack of in-depth experiments for N* variant. We have added additional discussion to point out that we performed additional biochemical experiments, as the reviewer suggested, to confirm that these mutations indeed lower N protein phosphorylation (Fig 1C and 1D) without affecting protein expression levels (S1A Fig). We have not been able to reproduce the PhosTag gel findings from the other studies. We disagree with the reviewer that our GSK-3 inhibition data is “complicated by effects of cytotoxicity” as we have performed a first systematic test of several kinase inhibitors (S2 Fig) and found GSK-3 inhibition to increase VLP assembly of low-assembly N variants at non-cytotoxic levels. In addition, given that the phenotype is increased assembly, we do not attribute this to cell death as that would have decreased VLP assembly. With regards to N*, we have demonstrated that N* can package and assemble into VLPs, it is well expressed and it is enriched in VLPs relative to full-length N protein. We have also cited our recent paper with the Morgan lab where we did more mechanistic studies on N* packaging (PMID: 37863261). We believe that additional mechanistic studies about N* are out of the scope of this paper.

**Fig. 1C and 1D**

**Fig. S1A**

**Fig. S2 GSK3 inhibitors**

**Part II – Major Issues: Key Experiments Required for Acceptance**

**Reviewer #1:**

1.The abstract is quite vague regarding the main findings, and their importance regarding the recent variants.

We have modified the abstract to be less vague and include more information about the variants as suggested by the reviewer.

2.The main text is also imprecise regarding which variants were used. For instance, page 4 bottom “we expressed and purified four SARS-CoV-2 N protein variants shown to enhance viral assembly”. The authors should indicate more clearly which variants they tested. They could also validate some of the results with recent variants. How is phosphorylated the N protein from the JN.1 lineage?

We have modified the text at lines 93, 124, 134, and 141 to be more clear about which variants were used. We have included the Omicron variant (N:R203K/G204R) as part of the study and those mutations still represent recent variants like JN.1 in the SR motif of the central linker region of N protein.

3.The authors show the phosphorylation state of N in transfected cells. Is it similar to that observed in infected cells?

We thank the reviewer for their suggestion. We measured the phosphorylation status of N protein in transfected cells as this is where the VLP and SCIP assays were conducted. We have attempted to isolate N protein from infected cells but did not observe sufficient expression and purity levels to analyze its phosphorylation status by Western blot.

**Reviewer #2:**

1. Is the observation consistent with other Sarbecoviruses, such as those detected in bats and pangolins, and also betacoronaviruses, such as MERS-CoV? Including at least other sarbecoviruses could significantly improve the significance of the current study. Is the degree of phosphorylation in N protein a factor contributing to the differences in disease severity between SARS-CoV-2 and SARS-CoV-1?

We thank the reviewer for their suggestion. We have assessed the roles of N protein phosphorylation in the assembly of SARS-CoV-1 vs SARS-CoV-2 and found them to be highly similar (Fig 2C). SARS-CoV-1 is more distant (89%, now indicated on line 144) than pangolin and SARS-related coronaviruses such as BANAL viruses and RaTG13, and therefore we predict those viruses to have a similar phosphorylation phenotype. We believe that investigation of the effect of N protein phosphorylation in other coronaviruses is out of the scope of this study and will be investigated in a future project.

**Fig. 2C**

2. I wondered why the author generated a spike-competent, N-deleted replicon system instead of both spike and N-deleted replicon system. A spike-deficient replicon system will only replicate a single round and might be the best model to study replication.

We thank the reviewer for their suggestion and agree that including a Spike-deficient replicon would be a good model to study single round replication. We have assessed the impact of GSK-3 inhibition on Spike-deficient replicon replication in several cell lines (Fig 3E-H) and found that replication decreased with increasing concentrations of CHIR98014. These data indicate that lower phosphorylation results in lower RNA replication levels. This finding is in line with the data we obtained using the N-deficient replicon as well. We attempted during the review period to rescue a Spike- and N-deficient replicon system but failed to produce reliable levels of replication to include in the study.

**Fig. 3E-H**

3. What are the underlying factors of not including omicron variants (R203K + G204R) in your fitness study? I suggest including this as two recent studies (As highlighted in your discussion) demonstrated a different observation.

We selected variants with significant differences in phosphorylation status in human cells (Fig. 1E) with low (R203M, S202R, P199L) and high (G204R and M234I) for the fitness study. We have indicated this now in the text starting on line 124. The Omicron mutations (R203K/G204R) showed an intermediate decrease in phosphorylation and were excluded from further analysis at this point of the study. We previously studied the Omicron mutations in structural proteins and found that these mutations enhance viral particle assembly (PMID: 35858386). We have now cited this paper and addressed the differences between observations in the discussion starting at line 363.

**Fig. 1E**

4. I was wondering why there is no increase in viral titers in Calu cells after R203M or S203R substitution at the N of the WA1 infectious clone. This work mainly uses cell lines and might not represent actual viral pathogenesis in vivo. It might be good to validate the results with organoid systems, or with animal model.

We thank the reviewer for their suggestion to utilize more complex models. We believe that this is out of the scope of this study. We have demonstrated the phenotype in several cell lines throughout the study including BHK-21-ACE2, VeroE6-TMPRSS2, VeroE6-ACE2/TMPRSS2, 293T-ACE2/TMPRSS2, HuH7.5-ACE2/TMPRSS2, and A549-ACE2. In the Johnson et al study that demonstrated enhanced replication of the R203K/G204R mutant, a different clone of Calu3 cells was used (i.e. Calu3 2b4). The different source of cells may result in different levels of kinase expression and consequently affect the phenotype of the mutants.

5. N phosphorylation could be one of the factors but might not be the only factor determining the replication fitness of the SARS-related coronaviruses. Other factors, such as receptor usage, entry efficiency, and degree of immune evasiveness, could also determine the risk of pre-pandemic SARS-related coronavirus.

We thank the reviewer for pointing this out. In addition to discussing the limitations of our study in the discussion section, we have added that N phosphorylation is probably not the only factor determining replication fitness of SARSr coronaviruses starting on line 367.

**Reviewer #3**:

It is surprising that statistical analyses are lacking for most subfigures. The number of independent replicates is also not stated for most experiments, so it is not clear how rigorous the experiments are for these studies. For Fig. 4D, n=1 is not sufficient.

We thank the reviewer for pointing this out. We have updated all figures and figure legends and associated statistical analyses. For almost all experiments, the data were representative of at least three to four independent experiments. For Fig 4D, this was a pilot experiment to help us determine the optimal ratio for the subsequent competition experiments. This has now been indicated in the figure legend.

Two other studies also characterized the impact of enhanced phosphorylation of N protein with the evaluation of the S202R and R203K/G204R mutants (PMID: 36572560 and 35728038). Findings here shown in Fig. 1 is opposite for some of their mutants. Stating that, “Both studies used PhosTag gels to evaluate phosphorylation, but this approach produced broad, difficult to interpret smears in our experiments due to the high degree of phosphorylation in N protein. The mutation at S202R in particular is unlikely to increase phosphorylation since it should block GSK3 phosphorylation at sites 202, 198, 194, 190 and 186” does not provide a satisfactory explanation or sufficient evidence. Orthogonal approaches to assess and confirm phosphorylation should be performed given the discrepancy of results.

We have now updated the discussion section to better address the discrepancy starting on line 359. We point out that in our hands we have not been able to resolve bands on the PhosTag gels and therefore cannot compare our findings directly to these studies. However, when using an orthogonal biochemical assay (Fig 1C and 1D) without affecting protein expression levels (S1A Fig), we find that S202R and R203K/G204R mutants indeed have lower phosphorylation levels. In the Johnson et al study, the authors have opposite results for the R203K/G204R mutant in Calu3 vs Vero cells using PhosTag gels and therefore we are not confident that this mutant consistently enhances phosphorylation of N protein. In addition, the inhibitor used in that study is not a GSK-3 specific inhibitor and therefore we think that comparison of the results between the two studies is not straightforward. In terms of fitness, we have cited our previous papers which agree with these studies that the S202R and R203K/G204R mutations improve assembly.

**Fig. 1C and 1D**

**Fig. S1A**

Fig. 2C, the authors make the comparison to SARS-CoV-1 N. How conserved is the linker sequence between CoV-2 N and CoV-1 N? Why was the comparison done with CHIR98014 and not the more specific SB216763 as in Fig. 2B?

We have now indicated in the text at line 143 that SARS-CoV-1 and SARS-CoV-2 share 89% sequence identity in the SR-rich motif of the central linker region of N protein. We have also now indicated in the text starting at line 141 that we systematically tested kinase inhibitors and chose CHIR98014 for the remainder of the study given it is more potent in enhancing the assembly of low-assembly N variants (see S2 Fig) at non-cytotoxic concentrations. We have not found data that SB216763 is more specific than CHIR98014 for GSK3, but CHIR98014 is more potent.

**Fig. S2 GSK3 inhibitors**

It is not clear how chimeric N protein variants generated by swapping sequences with MHV were validated and shown not to be “interfering with other N protein functions”. In this study, a luciferase readout was used to assess their impact in viral assembly and replication. No additional evaluation of “other vital N protein functions” were performed.

We thank the reviewer for pointing this out. We have revised the text starting on line 187 to indicate that we have validated that the N-D5 chimeric N protein does not support particle assembly (S3C Fig) and therefore is an appropriate N protein to use in the N-replicon single round assay as shown in S3A Fig. We took out the text regarding “other vital N protein functions” as we have only looked at assembly and replication and not any other functions. While we used the chimeric N-D5 protein to test the effect of mutations in single-round infections, we validated our data using an orthogonal S-deficient replicon in Fig 3E-H.

**Fig. S3A-S3C**

**Fig. 3E-H**

The authors developed the SCIP system but did not use it to test their N mutants of interest. Why? If the system uncouples entry from replication, it would have strengthened their results to see assays with trans-complemented mutant N proteins.

We have used the chimeric N-D5 protein which fully uncouples replication from assembly as validated in S3A Fig to test the effect of N protein mutations on replication. The data in Fig 3B and 3C show N trans-complementation assays that demonstrate the impact on replication of several low- and high-assembly N protein variants.

**Fig. 3B and 3C**

Fig. 3B, what is the rationale for using CHIR98014 and not SB216763? Based on Fig. S2, viability significantly decreases at CHI98014 concentrations >1 uM in 293T cells. This may be reflected in other cell types shown in Fig. 3D-H (cell viability for those cell types are not shown) and not reduced replication.

We have now indicated in the text starting at line 141 that we systematically tested kinase inhibitors and chose CHIR98014 for the remainder of the study given it is more potent in enhancing the assembly of low-assembly N variants (see S2 Fig) at non-cytotoxic concentrations compared with SB216763. We thank the reviewer for pointing out the omission of viability data for the cells indicated in Fig. 3D-H. We have added these data in S3F and did not observe cytotoxicity except for the 8 uM CHIR98014 dose. The effect we observe for inhibition of replication is at concentrations well below 8 uM (Fig 3E-H).

**Fig. S2 GSK3 inhibitors**

**Fig. S3F**

**Fig. 3E-H**

Evaluation of a role for N* is potentially interesting. However, the studies here are very limited. Fig. 5C does not show protein expression levels of truncated N-variants; lack of stable expression can account for lack of luciferase activity in packaging. What is the N* expression levels in VLPs? Western blot data should be shown along with the quantitation. With the VLPs, it is not clear what the N* expression levels are relative to N wt. Studies here do not examine the impact of N* on the multiple roles of N in viral replication and pathogenesis.

We agree with the reviewer that N* is an interesting avenue for SARS-CoV-2 to resolve the opposing phenotypes of N protein phosphorylation on particle assembly and RNA replication. We have used the experiment in Fig. 5C as a pilot to test truncations throughout the N protein and do not have full expression data for all the variants. We only selected positive truncation variants for further analysis. We have shown that N* is expressed well and is enriched in VLPs using Western blotting (S4 Fig) and quantification shown in Fig 5D. We have also cited our recent paper with the Morgan group showing that N* indeed is capable of packaging (PMID: 37863261). We believe that additional mechanistic studies are out of scope for this paper.

**Fig. 5C**

**Fig. S4**

**Fig. 5D**

**Part III – Minor Issues: Editorial and Data Presentation Modifications**
Reviewer #1: (No Response)

Reviewer #2: 1. Be consistent with the graph y-axis, and it might be good to display the graph in a log scale.

We thank the reviewer for their input. We have now changed the y-axes to be more consistent.

Reviewer #3: Fig 3B legend, missing description.
Fig. S3, times on images can be made clearer.
Fig. 4E-G, no drug data can be made darker.

We thank the reviewer for pointing these out. They have been corrected in the revised manuscript.
